# Comparison of electrical bioimpedance in newborns with electrodes positioned on the right and left sides of the body

**DOI:** 10.1016/j.jped.2025.03.007

**Published:** 2025-04-26

**Authors:** Thatyana Ribeiro Medeiros, Camila Barros Melgaço da Silva, Flávia Nunes Benicio de Souza, Hannah Schmidel Kautsky, Luana Martins de Oliveira, Luana Martins de Toledo, Alan Araújo Vieira

**Affiliations:** Universidade Federal Fluminense, Faculdade de Medicina, Departamento Materno Infantil, Niterói, RJ, Brazil

**Keywords:** Body composition, Electrical impedance, Newborn

## Abstract

**Objective:**

Bioelectrical impedance analysis is a method used to assess body composition; a noninvasive test performed using an easy-to-handle portable device used in clinical practice. However, nonstandard methods in neonates hinder external validation and reliability. Currently, bioimpedance analysis is performed in newborns with electrodes positioned on the right side of the body; however, the use of medical devices, including vascular access, can prevent its use.

**Methods:**

An uncontrolled before-after clinical trial comparing resistance and reactance measurements by bioelectrical impedance analysis on both sides was conducted. Measurements were performed immediately after the randomization of the initial measurement side. The sample size was calculated by considering a 10% deviation from the mean resistance and reactance values of previous studies with alpha and beta errors of 10% and 20%, respectively. Binary linear regression was used to quantify the correlation.

**Results:**

A significant difference was observed between resistance (672.88 ± 136.30 vs. 649.22 ± 119.59) and reactance (46.34 ± 17.99 vs. 44.439 ± 19.42) values measured on the right and left sides, respectively. However, when measured on both sides of the body, resistance and reactance values showed a good correlation (0.98 for both models, *p* < 0.001). Positioning the electrodes on the left side significantly affected the resistance and reactance values measured by bioelectrical impedance analysis compared with those on the right side.

**Conclusion:**

Electrodes positioned on opposite sides of the body generated different resistance and reactance values, implying the need to use the right side exclusively for standard positioning. This restriction can create difficulties for the routine use of this technique in newborns.

## Introduction

Assessing nutritional adequacy in newborns is essential because inadequate nutritional management in the early stages of life has long-term repercussions; however, monitoring nutritional adequacy can be challenging.^1^^,^^2^ Monitoring the quality of weight gain using body composition measurements can help understand the growth and nutritional adequacy of newborn infants.^1^^,^^3^^,^^4^

Among the methods used to assess body composition, bioelectrical impedance analysis is a low-cost, noninvasive, painless, practical, and safe procedure that can be easily performed at the bedside and repeated whenever necessary; indirectly assesses the amount of total body water.^5^ Bioelectrical impedance analysis runs an electric current through the body to measure its resistance and reactance and based on these measurements, indirectly calculates the body fluid distribution in the intra- and extracellular spaces, the cell membrane quality, size, and integrity.^2^^,^^4^^,^^6^ Bioimpedance in newborns is very suitable for measuring body water, but unfortunately, it does not provide results for other indices related to body composition, such as lean or fat-free mass and fat mass.^6^

Currently, in addition to the limited data on bioelectrical impedance analysis for newborn infants, there is no consensus on the methodological standard for this test in the pediatric population.^7^^,^^8^ In adults, the electrodes are positioned on the right hand and foot.^6^^,^^9^^,^^10^ However, the presence of vascular access, monitoring, and supporting equipment on the right side prevents bioelectrical impedance analysis in critically ill children.^8^^,^^11^ This study aimed to compare resistance and reactance values measured using bioelectrical impedance analysis with electrodes positioned on opposite sides of the body (right or left) in newborn infants.

## Methods

An uncontrolled before-after clinical trial comparing resistance and reactance measurements using bioelectrical impedance analysis with electrodes positioned on the right and left hands and feet of newborn infants. Measurements were taken immediately after randomization of the initial measurement side. The research protocol was approved by the Research Ethics Committee of Federal Fluminense University - FM/UFF (approval number 93,549,618.8.00005243) and was conducted in accordance with the tenets of the Declaration of Helsinki.

The standardized test for the adult population was adapted for use in newborn infants as follows: the internal arm electrode (red detector) was placed on the dorsal surface of the right wrist, between the ulnar and radial bones; the external electrode (black emitter) was placed on the third metacarpal bone; the internal leg electrode was placed on the anterior surface of the ankle, between the prominent portions of the bones; and the external electrode was placed on the surface of the third metatarsal bone. A minimum distance of 5 cm between electrodes was recommended for this procedure.

During the tests, neither the examiner nor the guardian touched the newborn infant, who was placed in the supine position, with the limbs kept away from the body or metal surfaces to avoid random dispersion of the electric current. The test lasted for approximately 5 min, and was performed 1.5 h after feeding to prevent emesis or interference with digestion when handling the NB. Measurements were not performed when the newborn infant was agitated or at an abnormal temperature. Newborn infants were carefully observed during the tests to detect any clinical changes that could interfere with their well-being as soon as possible.

The resistance and reactance values were measured using a Quantum 101Q single-frequency bioelectrical impedance analysis device (RJL Systems, USA), which applies a sinusoidal alternating current of 50 kHz and 800 μA. The device was calibrated according to the manufacturer's specifications every 20 assessments.

The tests were conducted in the neonatal unit of the university hospital. The inclusion criteria were full-term and premature newborns of both sexes. The exclusion criteria were critically ill newborns, discontinuous skin integrity at the electrode placement site, and the use of invasive treatment devices such as vascular access. Those responsible for the eligible newborns signed the consent statement.

Sample size calculation considered a 10% deviation from the mean resistance and reactance values from previous studies (60 and 5 ohms, respectively), an alpha error of 10%, and a beta error of 20%. The calculated sample size included 53 resistance and 203 reactance measurements.

The studied variables were represented as measures of central tendency and the means were compared using a paired *t*-test. Binary linear regression was performed by forcing the intercept to zero, with resistance and reactance measured on the right side as independent variables. Linear regression was used to assess the correlation between the resistance and reactance values measured on the right and left sides, and Bland-Altman scatter plots were plotted. The data were analyzed using R statistical and SPSS 16.0 software, at a 5% significance level.

## Results

In a crossover study, the same measurement was taken twice on the same participant at practically the same time (one measurement in immediate sequence to the other), eliminating the possibility that the participant interfered with the results, regardless of the sex of the newborn infants, gestational age (GA), weight, or any other characteristic assessed, because the participant was its own control. Table 1 shows the characteristics of the study population.

**Table 1:** Characteristics of newborn infants undergoing reactance (*n* = 203) and resistance (*n* = 53) measurements.

Variables	Reactance (*n* = 203)		Resistance (*n* = 53)	
	Mean ± SD	Min. and max. value	Mean ± SD	Min. and max. value
Age (days)	12 ± 6.0	1-27	14 ± 6.6	4-27
Weight (grams)	2190 ± 805	885-3775	1861 ± 630	1200-3475
GA (weeks)	34 ± 3.0	29-41	32 ± 2.6	29-41

SD, standard deviation; min, minimum; max, maximum; GA, gestational age.

Table 2 shows resistance and reactance values measured using a single-frequency bioelectrical impedance analysis device with electrodes positioned on the right and left sides of the newborn infants. A significant difference was observed between the resistance and reactance values measured on the right and left sides.

**Table 2:** Resistance and reactance values were measured using a single-frequency bioelectrical impedance analysis device with electrodes positioned on the right and left sides of the newborn infants.

	Right side	Left side		*p*-value*
	Mean ± SD	Mean ± SD	Difference between means	
Resistance Ω	672.88 ± 136.30	649.22 ± 119.59	23.66	0.028
Reactance Ω	46.34 ± 17.99	44.43 ± 19.42	1.91	0.044

Ω, ohms; SD, standard deviation; * paired *t*-test.

**Figure 1:**
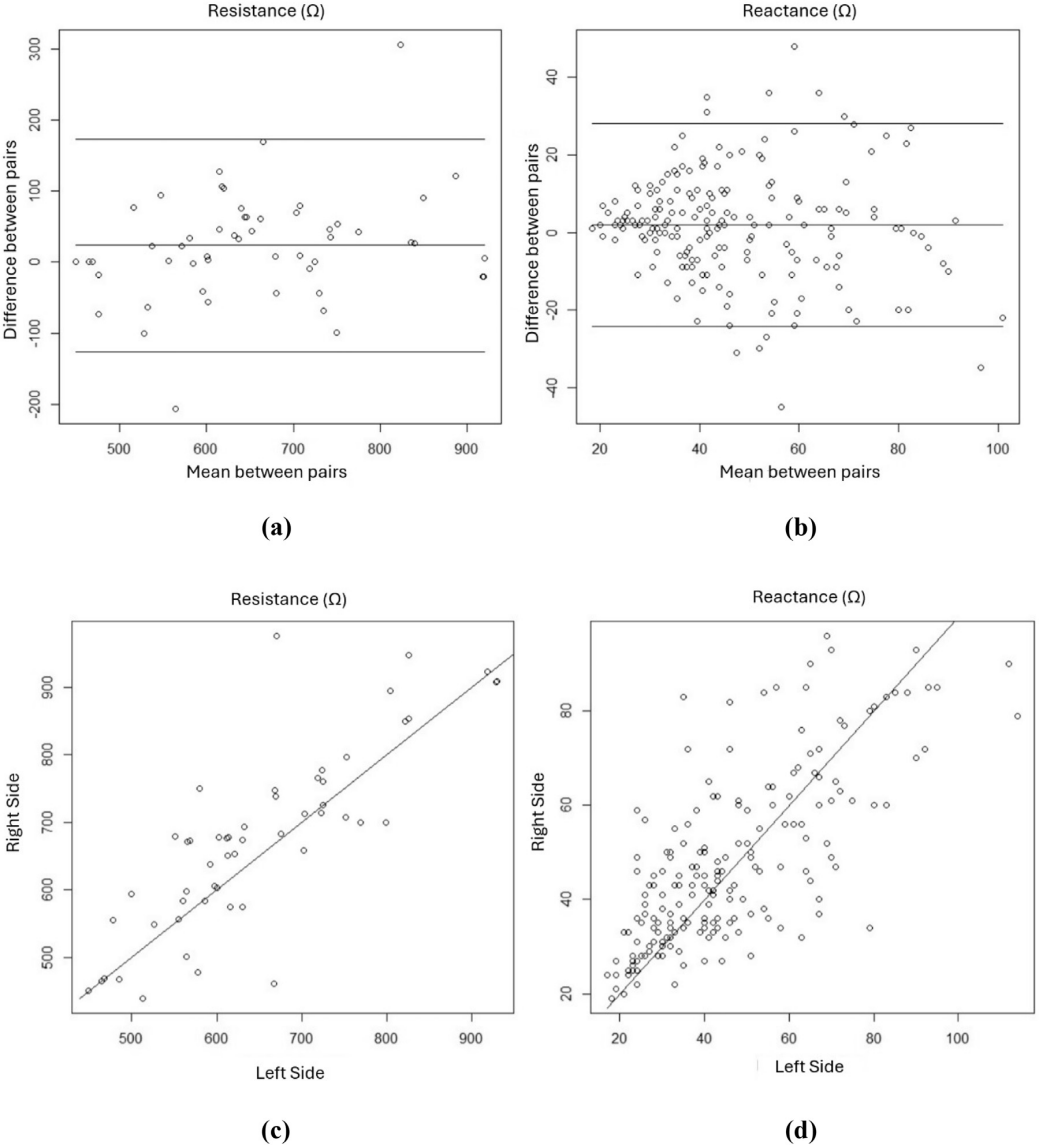
Bland-Altman scatter plots (Figure 1a and b) and linear regression plots (Figure 1c and d) for resistance and reactance measurements using bioelectrical impedance analysis with electrodes positioned on the right and left sides.

Figure 1 shows Bland-Altman scatter plots (Figure 1a and b) and linear regression plots (Figure 1c and d) for resistance and reactance measurements using bioelectrical impedance analysis with electrodes positioned on the right and left sides. The Bland-Altman plots for resistance measurements on the right and left sides (Figure 1a and b) showed differences between the means, mostly within the defined confidence interval. For reactance, the graph (Figure 1b) showed a progressively greater dispersion between the measured pairs with higher values. Linear regression plots for the resistance and reactance values from the right and left sides (Figure 1c and d) showed a good correlation between the measurements (r-fit = 0.987 and 0.926 for resistance and reactance, respectively, *p* < 0.001).

## Discussion

This study clearly demonstrated that, unlike the standardized method for adults, positioning the electrodes on the left side of the body for bioelectrical impedance analysis in newborns generated different resistance and reactance results, which prevented replacing the right side with the left side for bioelectrical impedance analysis. However, the measurements showed an excellent correlation.

The clinical applicability of total body water measurement in newborn infants is indisputable, especially in critically ill premature newborn patients who are more susceptible to developing pathologies associated with excessive fluid administration, such as bronchopulmonary dysplasia and patent ductus arteriosus, or with hypovolemia, such as arterial hypotension and metabolic acidosis.^2^^,^^5^^,^^12^ Therefore, the possibility of using bioimpedance repeatedly allows for strict control of total body water, which is essential for clinical management.^12^

However, the internal and external validity of bioelectrical impedance analysis using the currently recommended standardization for adults can generate uncertainty. Another aggravating factor is that critically ill newborn infants often require invasive monitoring and treatment devices, which can hinder resistance and reactance measurements on the right side using bioelectrical impedance analysis.

Determination of total body water, body compartment volume, phase angle, and bioelectrical impedance vector analysis add to the arsenal of tests readily available at the bedside to help neonatologists in their clinical decisions.^13^, ^14^, ^15^

Furthermore, these data are crucial for this population because water homeostasis is not yet fully understood.^2^ Therefore, finding scientific evidence that bioelectrical impedance analysis can be used in neonatal intensive care units is a major step forward.

There have been a few studies and publications on bioelectrical impedance analysis during the neonatal period.^5^^,^^7^^,^^8^ Prediction equations for TBW, extracellular water, and fat-free mass were initially developed for adults and then extrapolated to the pediatric and neonatal populations.^16^^,^^17^ However, these populations differ physiologically and anatomically.

Further studies with optimal internal and external validations are necessary to prove the possibility of using this technology in the neonatal population. Uncertainties regarding the ideal method for measuring resistance and reactance values in pediatric and neonatal populations prevent the definition of a single standard for bioelectrical impedance analysis in newborn infants, which will improve the interpretation of the results obtained.

Determining whether electrode positioning on the left side affects the resistance and reactance measurements is a major limitation to the use of this method because it affects the total body water, phase angle, and bioelectrical impedance vector analysis results in newborn infants, especially when they are critically ill and require several devices for clinical stabilization. Phase angle and bioelectrical impedance vector analysis calculations require multifrequency devices, which were not used in this study.

Considering that the equation used to calculate total body water[16] includes two anthropometric measurements (weight and foot length) and that resistance measurement by bioelectrical impedance analysis requires electrode positioning on the right side, this study clearly shows the impracticality of using single-frequency bioelectrical impedance analysis in newborn infants who are ill and require treatment devices on this side, such as a peripherally inserted central catheter or vascular dissections.

The conclusions of this study cannot be extrapolated to other tests with methodology already validated in the literature that use bioelectrical impedance analysis to determine clinical parameters related to the amount of body fluids, in relation to the predetermined body position of the electrodes. The results of this study are a warning and can be used as a basis for discussion for other researchers who understand that changing the positioning of the electrodes would be interesting, in some way, for their patients. In this case, I believe that they should review the test methodology in the same way that was evaluated in this study.

The resistance and reactance values obtained with bioelectrical impedance analysis electrodes positioned on the right side of the newborn infants differed from those measured with electrodes positioned on the left side. Further studies are required to standardize bioelectrical impedance analysis for neonatal populations.

## Conflicts of interest

The authors declare no conflicts of interest.
